# Antibody-drug conjugates in gynecologic malignancies: breakthroughs, challenges, and future directions

**DOI:** 10.3389/fimmu.2026.1831465

**Published:** 2026-06-15

**Authors:** Yue Shen, Shaojuan Li, Yuting Guo, Zhonghua Su, Jiayu Xu, Hua Chang

**Affiliations:** 1 Department of Gynecology, The First Hospital of China Medical University, Shenyang, Liaoning, China; 2 Department of Surgical Oncology and General Surgery, The First Hospital of China Medical University, Shenyang, Liaoning, China; 3 Department of VIP In-Patient Ward, The First Hospital of China Medical University, Shenyang, Liaoning, China

**Keywords:** antibody-drug conjugate (ADC), cervical cancer (CC), endometrial carcinoma (EC), gynecologic malignancies, ovarian cancer (OC)

## Abstract

Gynecologic malignancies — principally cervical cancer (CC), endometrial carcinoma (EC), and ovarian cancer (OC) — have been managed with conventional modalities including surgery, radiotherapy, chemotherapy, targeted therapy, and immunotherapy, yielding modest overall outcomes. The scarcity of effective later-line options has fueled rapid advances in antibody-drug conjugate (ADC) research. Precision-targeted, highly potent ADCs hold the promise of improving outcomes for patients with gynecologic tumors. To date, multiple ADCs directed against antigens such as tissue factor (TF), human epidermal growth factor receptor 2 (HER2), trophoblast cell-surface antigen 2 (Trop2), and folate receptor alpha (FRα) have entered clinical trials or received regulatory approval for CC, EC, and OC. These agents have demonstrated superiority over conventional chemotherapy in patients with recurrent or metastatic disease, with particularly pronounced efficacy in populations exhibiting high target expression. Nevertheless, clinical translation remains challenged by response heterogeneity attributable to tumor heterogeneity, the emergence of resistance mechanisms, and class-specific adverse events including ocular toxicity, interstitial lung disease (ILD), hepatotoxicity, and peripheral neuropathy. Continued refinement of next-generation ADC platforms — encompassing bispecific ADCs, dual-payload ADCs, peptide-drug conjugates (PDCs), and radionuclide-conjugated drugs — is anticipated to overcome current limitations and deliver superior oncologic outcomes.

## 1 Introduction

According to the most recent U.S. cancer statistics (1), an estimated 13,490 new cervical cancer (CC) cases and 4,200 deaths, 68,270 new endometrial carcinoma (EC) cases and 14,450 deaths, and 21,010 new ovarian cancer (OC) cases and 12,450 deaths are projected for 2026. Global cancer data indicate that CC ranks fourth in incidence among female malignancies (2), while EC has shown an increasing incidence with a trend toward younger patients in recent years, and OC carries the highest case-fatality rate among gynecologic malignancies. Despite improvements in early-detection screening and multimodal management over the past decade, the overall mortality of patients with advanced disease remains unacceptably high.

For gynecologic malignancies, conventional surgery, radiotherapy, and chemotherapy have been supplemented by targeted therapy and immunotherapy, which have demonstrated benefit in select subgroups; however, the overall efficacy remains limited (3) and defined resistance mechanisms (4) leave the majority of patients with advanced disease facing a paucity of later-line options. There is, therefore, an urgent clinical need for novel therapeutic strategies capable of delivering highly potent cytotoxic agents with precision while overcoming the limitations of conventional chemotherapy and targeted therapy resistance. Against this background, antibody-drug conjugates (ADCs) have emerged as a class of “guided-missile” therapeutics that link a tumor-targeting monoclonal antibody (mAb) to a highly cytotoxic small-molecule payload (approximately 100–1,000-fold more potent than conventional chemotherapy) via a cleavable or non-cleavable chemical linker (5). Compared with conventional chemotherapy, ADCs offer precision targeting, enhanced efficacy, and a relatively favorable safety profile, with the potential to improve outcomes in malignant disease.

This review provides an in-depth examination of the current status of clinical ADC research across the major gynecologic malignancies, alongside the challenges encountered and future development directions.

## 2 Overview of ADCs

The concept of ADCs was proposed as early as the beginning of the twentieth century, yet it was not until 2000 that the first U.S. Food and Drug Administration (FDA)-approved ADC — gemtuzumab ozogamicin, targeting CD33 for the treatment of acute myeloid leukemia — was introduced, representing a landmark milestone in ADC development (6). Subsequently, ADC progress was hampered by challenges relating to linker instability and off-target payload toxicity (7). It was not until 2011 that technological advances enabled the successive approvals of brentuximab vedotin (8) and trastuzumab emtansine (9). In recent years, the next-generation ADC trastuzumab deruxtecan (T-DXd) has leveraged a high drug-to-antibody ratio (DAR) and a potent “bystander effect” to achieve breakthrough efficacy across multiple solid tumors including breast and lung cancer (10, 11), driving an exponential expansion of the ADC pipeline. As of November 2025, 19 ADCs have received regulatory approval for the treatment of solid and hematologic malignancies worldwide, with more than 200 additional agents in various stages of clinical evaluation.

**Figure 1:**
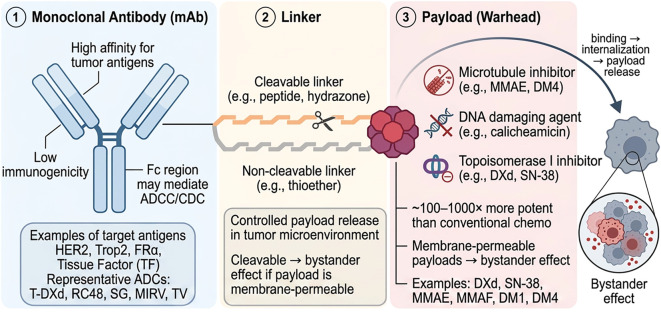
The main structure of the ADCs.

**Figure 2:**
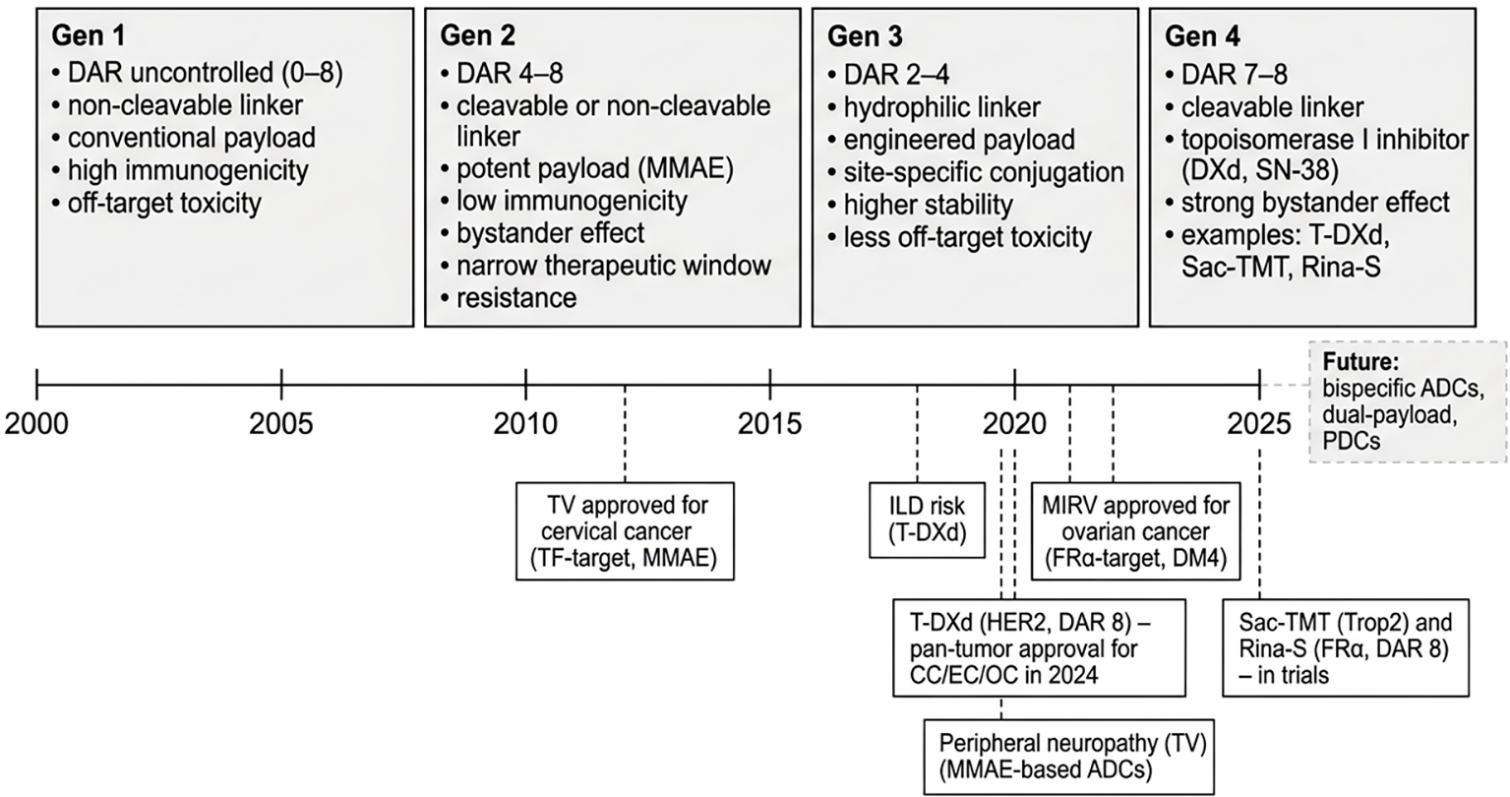
The timeline of ADCs development.

The core efficacy of an ADC originates from the precisely orchestrated synergy among its three constituent elements: antibody, linker, and cytotoxic payload. The main structure of the ADCs is shown in **Figure 1** and the timeline of ADC development is shown in **Figure 2**. The monoclonal antibody (mAb) serves as the “navigation system,” selectively recognizing and binding to antigens overexpressed on tumor cell surfaces to achieve targeted delivery. Accessible targets include tumor-overexpressed antigens or oncogene-encoded antigens — such as folate receptor alpha (FRα), trophoblast cell-surface antigen 2 (Trop2), prostate-specific membrane antigen (PSMA), mesothelin, CD56, CD70, Nectin4, human epidermal growth factor receptor 2 (HER2), and epidermal growth factor receptor (EGFR) (12) — as well as critical antigens within the tumor microenvironment, including vascular endothelial growth factor receptor 2 (VEGFR2) within the vasculature (13) and collagen IV within the stroma (14). The chemical linker functions as a critical “safety mechanism” (15): cleavable linkers (e.g., acid-labile hydrazone bonds, protease-sensitive peptide bonds) release the payload in response to tumor microenvironment-specific stimuli, and payloads with high membrane permeability can traverse cells to exert bystander effects; non-cleavable linkers (e.g., thioether bonds) release the payload only after antibody catabolism, offering greater stability but reduced bystander activity. The potent payload acts as the “warhead,” typically comprising highly effective cytotoxic agents targeting DNA or the microtubule network that can induce tumor cell apoptosis at extremely low concentrations (16, 17). In addition, certain membrane-permeable payloads are capable of penetrating cell membranes to generate a “bystander effect,” eliminating neighboring heterogeneous tumor cells (18).

The payloads of currently FDA-approved ADCs include: tubulin inhibitors (e.g., monomethyl auristatin E (MMAE), monomethyl auristatin F (MMAF), and the maytansine derivatives DM1 and DM4); DNA-damaging agents (e.g., calicheamicin and pyrrolobenzodiazepine dimers); and topoisomerase I inhibitors (e.g., SN-38 and DXd). Investigational payloads include tubulysins (19), duocarmycins (20), doxorubicin (21), and α-amanitin (22), as well as non-cytotoxic payloads such as stimulator of interferon genes (STING) agonists (23) and proteolysis-targeting chimera (PROTAC) molecules (24), representing highly promising next-generation warheads. We have summarized the ADC drugs in gynecological tumors in **Supplementary Table 1**.

## 3 Methods

A comprehensive literature search was conducted across PubMed/MEDLINE, Embase, Web of Science, and the Cochrane Library for articles published from January 2000 through December 2025. The search strategy combined subject headings and free-text terms related to antibody–drug conjugates (“antibody-drug conjugate,” “ADC,” “immunoconjugate”), gynecologic malignancies (“ovarian cancer (OC),” “cervical cancer (CC),” “endometrial cancer (EC),” “uterine cancer,” “fallopian tube cancer,” “primary peritoneal cancer”), relevant molecular targets (“FRα,” “HER2,” “Trop2,” “tissue factor (TF),” “B7-H4,” “CDH6,” “sodium-dependent phosphate transport protein 2B (NaPi-IIb),” “claudin 6,” “mesothelin”), and specific drug names [e.g., mirvetuximab soravtansine, trastuzumab deruxtecan (T-DXd), tisotumab vedotin (TV), sacituzumab govitecan (SG)]. Boolean operators (AND/OR) were applied to combine search terms, with syntax adapted to each database.

To supplement the electronic database search, we manually reviewed abstracts and presentations from major international oncology conferences held between 2020 and 2025, including the American Society of Clinical Oncology (ASCO) Annual Meeting, the European Society for Medical Oncology (ESMO) Congress, the Society of Gynecologic Oncology (SGO) Annual Meeting, and the American Association for Cancer Research (AACR) Annual Meeting. The ClinicalTrials.gov registry was searched systematically to identify ongoing or recently completed phase I–III trials of ADCs in gynecologic cancers. Regulatory documents, drug labels, and safety communications from the FDA and the European Medicines Agency (EMA) were also reviewed. Reference lists of included articles were hand-searched to capture additional relevant studies.

## 4 ADCs for cervical cancer

Despite recent advances in systemic therapy, the prognosis of recurrent or metastatic CC remains poor once resistance to standard chemotherapy or immunotherapy develops. ADCs have attracted increasing interest in CC because they combine tumor-specific targeting with highly active cytotoxic payloads, offering a potential means to improve efficacy while limiting off-target toxicity. We summarized the key clinical trial results in **Table 1**.

**Table 1:** Clinical trials results for cervical cancer.

Study name	Phase	Population	Study arms / design	Key efficacy results	Reference
innovaTV 301	Phase III	Recurrent or metastatic cervical cancer after 1–2 prior systemic regimens	Tisotumab vedotin vs investigator’s choice chemotherapy	HR 0.70 mPFS: 4.2 months vs. 2.9 months OS: 11.5 months vs. 9.5 months	(25)
innovaTV 205 / GOG-3024 / ENGOT-cx8	Phase Ib/II	Recurrent or metastatic cervical cancer	Multi-cohort: TV + carboplatin; TV + pembrolizumab; TV + bevacizumab	ORR: 38%∼55%	(26)
DESTINY-PanTumor02	Phase II	Histologically confirmed locally advanced, unresectable, or metastatic cervical cancer	Single-arm	ORR: 50.0%(HER2 3+: 75%) mPFS: 7.0 months mOS: 13.6 months	(3)
RC48-C018	Phase II	HER2-expressing second-line recurrent or metastatic cervical cancer	Single-arm	ORR: 6.4% mPFS: 4.37 months	(27)
SHR-A1811	Phase II	HER2-expressing or HER2-mutated advanced solid tumors, including possible gynecologic subgroups	Single-arm	ORR: 63.6% DCR: 93.9% PFS: 10.7 months	(28)

### 4.1 Tissue factor

Tissue factor (TF; also known as CD142) is a transmembrane glycoprotein encoded by the F3 gene that is specifically overexpressed across a range of solid tumors, including CC (93.3%–100%), OC (75%–100%), and EC (14%–100%) (29); its expression level correlates closely with tumor proliferation, angiogenesis, metastasis, and adverse prognosis. Tisotumab vedotin (TV), the first globally approved TF-targeted ADC, exerts multifaceted antitumor activity through MMAE-mediated cytotoxicity, antibody-mediated blockade of the TF–FVIIa signaling pathway, and Fc-region-dependent immune effector functions (30). TV (Tivdak^®^) received FDA accelerated approval in September 2021 for the treatment of adult patients with recurrent or metastatic CC with disease progression on or after chemotherapy (second line and beyond), making it the first ADC approved by the FDA for CC (30). In 2023, full FDA approval was granted based on the phase III innovaTV 301 data.

Multiple clinical studies have demonstrated the efficacy and safety of TV in recurrent or metastatic CC. In the phase III randomized trial innovaTV 301, TV was directly compared with investigator’s choice of chemotherapy. The results showed that the median overall survival (OS) was 11.5 months in the TV group versus 9.5 months in the chemotherapy group, corresponding to a 30% reduction in the risk of death (hazard ratio (HR) 0.70, 95% confidence interval (CI) 0.54–0.89), indicating a clinically meaningful survival benefit (25). Notably, although median progression-free survival (PFS) was also improved with TV compared with chemotherapy (4.2 months vs. 2.9 months), the absolute difference was relatively modest. This suggests that its benefit in delaying disease progression may be limited, and that the OS improvement may in part reflect a sustained post-progression treatment effect or the impact of subsequent therapies. Therefore, in clinical practice, both OS and PFS data should be considered to provide a comprehensive and balanced assessment of the therapeutic value of TV. In addition, the phase Ib/II innovaTV 205 study explored the feasibility of combining TV with carboplatin or pembrolizumab in both first-line and later-line settings. Preliminary results showed that these combination strategies achieved relatively high objective response rates (ORR, 38%–55%), suggesting the potential to further improve efficacy, although confirmation in larger prospective studies is still needed (26). At present, TV has been incorporated into the National Comprehensive Cancer Network (NCCN) Clinical Practice Guidelines in Oncology for CC as one of the preferred second-line or subsequent therapy options for recurrent/metastatic cervical cancer (category 1 evidence). The NCCN Guidelines also recommend the combination of TV plus pembrolizumab for programmed death-ligand 1 (PD-L1)-positive patients who have not previously received immunotherapy (31).

Beyond TV, additional TF-targeted ADCs — including MRG004A, XB002, and agents carrying topoisomerase inhibitor payloads such as XNW28012 and AMT-754 — are under active clinical development (32, 33). However, TF-targeted ADCs loaded with microtubule inhibitors (e.g., MMAE), such as TV, MRG004A, and XB002, still face challenges from specific toxic side effects including bleeding, ocular adverse reactions, and peripheral neuropathy (25). In contrast, TF-targeted ADCs carrying topoisomerase inhibitors (e.g., XNW28012, AMT-754) exhibit lower risks of peripheral neuropathy, but their overall safety profile requires further clinical data validation.

### 4.2 HER2

HER2, a receptor tyrosine kinase encoded by the ERBB2 gene, is closely associated with aggressive tumor biology including hyperproliferation, resistance to apoptosis, and metastasis when overexpressed (34, 35). Among gynecologic malignancies, HER2 expression is highest in EC (immunohistochemistry (IHC) 3+: 12.6%; IHC 2+: 38.6%), followed by OC (IHC 3+/2+: 14.3% each) and CC (IHC 3+: 4.7%; IHC 2+: 20%) (36).

T-DXd (DS-8201) is composed of a humanized anti-HER2 IgG1 antibody conjugated to the topoisomerase I inhibitor DXd via a cleavable tetrapeptide linker. Its key advantages reside in the high DAR (~8) and the strong membrane permeability of DXd, which confers a potent “bystander effect” (37). DESTINY-PanTumor02 (3) demonstrated that, among 40 patients with HER2-positive CC, the ORR was 50.0% (75% in the HER2 IHC 3+ subgroup), with a median PFS of 7.0 months and median OS of 13.6 months. NCCN guidelines recommend T-DXd for patients with HER2-positive (IHC 2+ or 3+) recurrent/metastatic CC (38). T-DXd (Enhertu^®^) received FDA accelerated approval in April 2024 — based on DESTINY-PanTumor02 data — for the treatment of adult patients with HER2-positive (IHC 3+) unresectable or metastatic CC (squamous cell carcinoma, adenocarcinoma, or adenosquamous carcinoma), representing both the first pan-tumor HER2-directed ADC approval and the first approved HER2-targeted ADC in CC (3).

Disitamab vedotin (RC-48), a Chinese-developed ADC composed of a humanized anti-HER2 antibody, a cleavable linker, and MMAE (DAR 4) (39), was evaluated in the RC48-C018 study (NCT04965519) (27), which enrolled patients with recurrent/metastatic CC who had progressed on ≥1 prior platinum-based regimen. The ORR reached 36.4%, with a median PFS of 4.37 months and a 12-month OS rate of 66%. The AK001 study conducted based on this demonstrated that when RC48 was combined with camidanilumab, the ORR increased to 50.0%, and the disease control rate (DCR) reached as high as 70.5%, indicating the synergistic potential of the “ADC combined with immunotherapy” strategy (40). Disitamab vedotin (Aidixi^®^) has not received FDA approval; it was approved in June 2021 by China’s National Medical Products Administration (NMPA) for the treatment of patients with HER2-overexpressing (IHC 2+ or 3+) locally advanced or metastatic gastric cancer (including gastroesophageal junction adenocarcinoma). Gynecologic oncology indications are currently under clinical investigation.

SHR-A1811 (regatrastuzumab deruxtecan) is another HER2-targeted ADC consisting of trastuzumab, a cleavable linker, and the topoisomerase I inhibitor (41). In a preliminary phase II study (NCT05896020), among 35 heavily pretreated patients with HER2-expressing advanced CC, the ORR reached 63.6%, with a median PFS of 10.7 months and a DCR of 93.9% (28). SHR-A1811 has not received FDA approval and is currently in phase II/III clinical evaluation.

### 4.3 Trop2

Trop2 is broadly expressed across epithelial gynecologic tumors, with expression rates of 88.7%, 72%, and 59% in CC, EC, and OC, respectively (42, 43).

Sacituzumab govitecan (SG) is composed of a humanized anti-Trop2 monoclonal antibody, an acid-labile cleavable linker, and SN-38 (44). The EVER-132–003 study demonstrated an ORR of 43% and a DCR of 85% in 40 patients with advanced recurrent CC who had progressed after platinum-based therapy, with a median PFS of 7.1 months; in the 27 patients with prior immunotherapy, the ORR was as high as 48% with a median PFS of 8.3 months (45). SG (Trodelvy^®^) has not received FDA approval for a cervical cancer indication.

Sacituzumab tirumotecan (Sac-TMT, MK-2870) comprises a humanized monoclonal antibody, a cleavable linker, and the topoisomerase I inhibitor KL610023 (46). In the SKB264-II-06 study (NCT05642780), Sac-TMT combined with pembrolizumab for the treatment of advanced CC yielded an ORR of 57.9%, a DCR of 86.8%, and a 6-month PFS rate of 65.7%, with both median PFS and median duration of response (DOR) not yet reached, highlighting the outstanding potential of this combination strategy.

MK-2870-001/KL264-01 (NCT04152499) (47) is a phase I/II clinical study, and the efficacy and safety results of single agent treatment with Sac-TMT for advanced/metastatic CC were announced at the 2025 ESMO conference. As of the data deadline (November 18, 2024), a total of 58 patients have received at least one dose of Sac-TMT monoclonal antibody. At the time of data cut-off, 24 patients (41%) were still receiving treatment. The confirmed ORR is 28% (95% CI: 17-41), and the confirmed+unconfirmed ORR is 38% (95% CI: 26-52); The median DOR has not yet been reached. The median PFS was 6.1 months (95% CI: 3.9- not achieved). MK-2870-001/KL264-01 (NCT04152499) (48) is a phase I randomized, actively controlled, open label, multicenter study comparing the efficacy and safety of Sac-TMT monotherapy and physician choice therapy as second-line treatment for recurrent or metastatic CC. The results will provide important evidence for the application of MK-2870 in recurrent or metastatic CC. Sac-TMT has not received FDA approval and is currently in phase III evaluation (TroFuse series).

### 4.4 Other antigens

In addition to TF, HER2, and TROP2, several other ADC targets are being explored in CC, although most remain in the preclinical or early clinical stage.

Another potential target is mesothelin, a cell-surface glycoprotein overexpressed in several solid tumors. Mesothelin-directed ADCs, such as anetumab ravtansine, have shown preliminary antitumor activity in mesothelin-expressing malignancies, but clinical evidence in CC is currently very limited, and no mature CC-specific efficacy results have been formally established (49, 50).

AXL receptor tyrosine kinase (AXL) has also been investigated as a therapeutic target in solid tumors because of its association with invasion, metastasis, and treatment resistance. Experimental ADCs targeting AXL, such as enapotamab vedotin, have entered early-phase studies in advanced solid tumors; however, published cervical cancer-specific outcomes remain unavailable, and its role in CC is therefore still exploratory (51, 52).

In addition, targets such as B7-H3 (CD276) are receiving increasing attention because of their broad expression in solid tumors and immunoregulatory functions. Several B7-H3-directed ADCs, including ifinatamab deruxtecan (DS-7300) and other investigational agents, are undergoing early clinical evaluation in solid tumors, but there is currently no published mature dataset dedicated to CC (53, 54). Overall, beyond TF, HER2, and TROP2, ADC development in CC is still at an early stage, and most alternative targets have not yet generated disease-specific clinical evidence sufficient to define their therapeutic value.

## 5 ADCs for endometrial cancer

EC is the most common gynecologic malignancy in developed countries, and patients with recurrent or metastatic disease have limited treatment options once resistance to platinum-based chemotherapy and immune checkpoint inhibitors develops. In recent years, ADCs have emerged as a promising therapeutic strategy in EC by combining selective tumor targeting with highly potent cytotoxic payloads, particularly in biomarker-defined populations. We summarized the key clinical trial results in **Table 2**.

**Table 2:** Clinical trials results for endometrial cancer.

Study name	Phase	Population	Study arms / design	Key efficacy results	Reference
DESTINY-PanTumor02	Phase II	HER2-expressing advanced solid tumors, including endometrial cancer	Single-arm, multi-cohort	ORR: 57.5%(HER2 3+: 84.6%) mPFS: 11.1 months	(3)
KL264-01	Phase II	Endometrial cancer	Single-arm cohort	ORR: 34.1%	(47)
IMMU-132-01	Phase I/II	Endometrial cancer	Single-arm	ORR: 22.2%, mPFS: 3.2 months mOS: 11.9 months	(55)
RAINFOL-01	Phase I/II	Endometrial cancer	Single-arm	ORR: 50%	(56)

### 5.1 HER2

Among the three major gynecologic malignancies, EC exhibits the highest frequency of HER2 protein overexpression. The DESTINY-PanTumor02 study (3) confirmed the activity of T-DXd in HER2-expressing EC. Among 40 HER2-positive patients, the ORR was 57.5% overall, with rates of 84.6% in the IHC 3+ subgroup and 47.1% in the IHC 2+ subgroup; the median PFS was 11.1 months and the median OS was 26.0 months. These results underscore the particularly striking efficacy of T-DXd in patients with high HER2 expression (IHC 3+). Clinical trial NCT05150691 (61) is designed to assess the safety, tolerability, pharmacokinetics, and preliminary anti-tumor efficacy of DB-1303/BNT323 in patients with advanced or metastatic solid tumors. Recruitment for this trial is currently underway.

It is noteworthy that the over-expression rate of HER2 in uterine carcinosarcoma is approximately 15% to 20%, and this population is also significant for T-DXd treatment. A phase II study (STATIONE trial) incorporated 32 patients with recurrent uterine carcinosarcoma who had previously experienced chemotherapy failure. The findings indicated that the ORR of the HER2 high-expression group (IHC ≥ 2+, n = 22) evaluated by the central laboratory was 54.5%, while the ORR of the HER2 low-expression group (IHC 1+, n = 10) was 70.0%. The median PFS was 6.2 months and 6.7 months respectively, and the median OS was 13.3 months and below (59, 62). NCCN guidelines have also listed HER2 IHC 1+/2+/3+ uterine carcinosarcoma as a recommended indication (38). T-DXd (Enhertu^®^) received FDA accelerated approval in August 2024 for an expanded indication encompassing HER2-positive (IHC 3+) unresectable or metastatic EC (including uterine carcinosarcoma) in adult patients with prior systemic therapy (3).

### 5.2 Trop2

Trop2 exhibits high expression levels in EC. Research findings have indicated that approximately 70%-80% of EC tissues demonstrate moderate to high expression of TROP2, which offers a biological foundation for the utilization of TROP2-targeted ADCs in this particular tumor.

Trop2 represents another highly expressed target in EC. A phase II study (KL264-01, Sac-TMT EC cohort) demonstrated an ORR of 34.1% with Sac-TMT in 44 patients with recurrent EC; notably, the ORR reached 47.7% in patients who had received only one prior line of therapy and 52.3% in those with two or more prior lines (63). A separate phase I/II basket study (IMMU-132-01; NCT01631552) reported an ORR of 22.2%, a median PFS of 3.2 months, and a median OS of 11.9 months in a subgroup of 18 patients with metastatic EC who had previously received platinum-based chemotherapy, providing preliminary validation of the feasibility of Trop2-targeted ADC therapy in this disease (55). Both SG and Sac-TMT have not received FDA approval for EC indications. Several large phase III trials are currently underway: ASCENT-GYN-01 (comparing SG versus physician’s choice) and TroFuse-005/MK-2870-005 (comparing Sac-TMT versus physician’s choice). The TroFuse-033 study is evaluating Sac-TMT combined with pembrolizumab as first-line maintenance therapy in patients with proficient mismatch repair (pMMR) EC. Datopotamab deruxtecan (Dato-DXd) is an ADC that targets Trop2, which is composed of anti-Trop2 monoclonal antibodies, cleavable linkers, and the topoisomerase I inhibitor DXd. Prior research has indicated that Dato-DXd has been incorporated into early clinical trials for multiple tumor types, including patients with EC, a type of solid tumor, as exemplified by the TROPION-PanTumor01 trial (NCT03401385) (64). Nevertheless, as of the present time, there are no well-established efficacy results for the EC cohort, and its clinical value necessitates validation through subsequent prospective studies. The field is witnessing a gradual paradigm shift from second-line treatment toward exploration of first-line maintenance strategies.

### 5.3 Other antigens

Beyond HER2 and Trop2, several additional ADC targets are being explored in EC, although the available evidence remains limited. FRα is one of the more promising candidates, as its expression has been reported in serous EC and in a subset of high-grade ECs, suggesting potential therapeutic relevance (65). The FRα-directed ADC mirvetuximab soravtansine (MIRV) has demonstrated clinical activity in OC; however, there are currently no mature, formally published EC-specific efficacy data to support its routine application in this disease (65).

Mesothelin is another potential target, given its overexpression in several solid tumors and its reported expression in subsets of aggressive ECs (66). Although mesothelin-targeting ADCs such as anetumab ravtansine have entered early-phase clinical development in mesothelin-positive solid tumors, published mature clinical data specifically in EC are still lacking (66).

In addition, B7-H3 (CD276) has emerged as a candidate target because of its broad expression and immunoregulatory role in solid tumors, including gynecologic malignancies (67). Several B7-H3-directed ADCs, such as ifinatamab deruxtecan (DS-7300), are currently being evaluated in early-phase solid tumor trials, but no dedicated mature dataset for EC has yet been reported (67). Overall, ADC development beyond HER2 and Trop2 in EC remains at an early stage, and further translational and clinical studies are needed to clarify the therapeutic value of these alternative targets.

B7-H4 (VTCN1) is a member of the B7 immune checkpoint family and is frequently overexpressed in EC. The positivity rate in different histological subtypes is about 50% to 80%, making it an attractive therapeutic target for ADC development. Currently, multiple B7-H4 targeted ADCs have been clinically studied in EC.

AZD8205 is a novel B7-H4 targeted ADC composed of a humanized anti-B7-H4 antibody coupled to a topoisomerase I inhibitor payload via a cleavable linker. A phase I/II, open label, multicenter study (NCT05123482) (68) is evaluating the use of AZD8205 in late stage or metastatic solid tumors (including EC) expressing B7-H4, with the main objective of determining recommended phase II doses, evaluating safety and tolerability, and preliminary anti-tumor activity. Early data released at the 2024 ASCO Annual Meeting showed that AZD8205 exhibited encouraging clinical activity and overall controllable safety features in patients with endometrial cancer undergoing multi line therapy. The Phase III clinical trial (NCT07044336) (69) is currently underway to evaluate the progression of B7-H4 late/metastatic EC between AZD8205 and physician selected chemotherapy (doxorubicin or paclitaxel) after platinum based chemotherapy and anti-PD-1/anti-PD-L1 treatment.

In addition, SGN-B7H4V is another B7-H4 targeted ADC developed by Seagen (now Pfizer), using MMAE as the payload and cleavable linker coupling via protease. A first human phase I study (NCT05194072) (70) is evaluating the safety, pharmacokinetics, and preliminary efficacy of SGN-B7H4V in advanced solid tumors, including B7-H4 expressing EC and OC.

On the other hand, HS-20089 is a B7-H4 targeted ADC developed by Hanson Pharmaceuticals, currently being evaluated for its application in locally advanced or metastatic solid tumors in a phase I/II clinical trial (NCT05263479) (71), which included the EC cohort. The preliminary results suggest that the drug has ideal anti-tumor activity and acceptable toxicity characteristics in B7-H4 positive gynecological tumors. At present, a randomized, open label, multicenter, phase III clinical study (NCT07286331) (72) with an EC cohort aims to compare the efficacy of GSK5733584 (HS-20089) and chemotherapy in patients. The study has not yet begun recruitment.

## 6 ADCs for ovarian cancer

Several ADCs targeting distinct surface antigens, including FRα, HER2, Trop2, and NaPi-IIb, have entered clinical investigation in OC. Among these, FRα-directed ADCs have shown the most mature clinical activity to date, whereas other targets remain under active evaluation in early-phase or disease-specific studies. We summarized the key clinical trial results in **Table 3**.

**Table 3:** Clinical trials results for ovarian cancer.

Study name	Phase	Population	Study arms / design	Key efficacy results	Reference
SORAYA	Pivotal single-arm study	FRα-high, platinum-resistant ovarian cancer; prior bevacizumab	Single-arm	ORR: 32.4% DOR: 6.9 months mOS: 13.8 months	(57)
MIRASOL	Phase III	FRα-positive platinum-resistant ovarian cancer	Mirvetuximab soravtansine vs investigator’s choice chemotherapy	ORR: 42.3% vs. 15.9% mPFS: 5.62 months vs. 3.98 months mOS: 16.46 months vs. 12.75 months HR 0.67	(58)
RAINFOL-01	Phase I/II	Ovarian cancer	Single-arm	ORR: 55.6%	(56)
DESTINY-PanTumor02	Phase II	HER2-expressing advanced solid tumors, including ovarian cancer	Single-arm, multi-cohort	ORR: 45%,(HER2 IHC 3+: 63.6%) mPFS:5.9 months mOS: 13.2 months	(3)
KL264-01	Phase II	Ovarian cancer progressing after prior platinum-based therapy	Single-agent sacituzumab tirumotecan	ORR: 40.0% DCR: 75.0% mPFS: 6.0 months	(59)
TROPION-PanTumor03	Phase II	High-grade serous or endometrioid ovarian cancer after 1–2 prior platinum-based regimens	Single-arm cohort	ORR: 42.9% DCR:75.0% mPFS: 5.6 months	(60)

### 6.1 FRα

FRα, a glycosylphosphatidylinositol-anchored cell-surface glycoprotein encoded by the FOLR1 gene, is widely overexpressed in high-grade serous OC and represents a critical driver of tumor cell proliferation, invasion, and metastasis. FRα-targeted ADCs under development primarily employ tubulin inhibitors or topoisomerase I inhibitors as their payloads. Among those utilizing tubulin inhibitors are MIRV (DAR 3.5), STRO-002 (DAR 4), MORAb-202 (DAR 4), and IMGN151 (DAR 3.5); topoisomerase I inhibitor-based agents include AZD5335, rinatabart sesutecan (Rina-S), BAT8005, LY4170156, and ZW191 (DAR predominantly 7–8).

MIRV is conjugated via a cleavable linker linking an anti-FRα monoclonal antibody to the tubulin inhibitor DM4. The pivotal phase III SORAYA study (NCT04296890) demonstrated an ORR of 32.4%, a median DOR of 6.9 months, and a median OS of 13.8 months in patients with FRα-high, platinum-resistant OC (57). The confirmatory phase III MIRASOL trial (NCT04209855) — enrolling patients with FRα-high (When using immunohistochemistry detection, ≥ 75% of surviving tumor cells exhibit at least moderate intensity ≥ 2+) membrane staining) platinum-resistant OC — showed that the MIRV arm achieved a significantly longer median PFS than chemotherapy (5.62 vs. 3.98 months; hazard ratio (HR) 0.65), a significantly higher ORR (42.3% vs. 15.9%), a significantly prolonged median OS (16.46 vs. 12.75 months; HR 0.67), and a superior safety profile (58). The NCCN Guidelines Version 3.2025 recommend MIRV monotherapy for patients with platinum-resistant OC with high FRα expression; for patients with low to medium FRα expression (≥25% but <75%), the combination with bevacizumab may be considered (73). MIRV received FDA accelerated approval in November 2022 for adult patients with FRα-high (PS2+ ≥75%), platinum-resistant OC, fallopian tube cancer, or primary peritoneal cancer after 1–3 prior lines of therapy, becoming the first ADC approved by the FDA for OC. Full FDA approval was subsequently granted in March 2024 based on the MIRASOL data, further consolidating its role as second-line therapy for OC.

Rina-S, a next-generation FRα-targeted ADC with a DAR of 8 and a topoisomerase I inhibitor payload, demonstrated an ORR of 50% in patients with advanced EC (100 mg/m² cohort) and an ORR of 55.6% with a DOR not yet reached in the OC dose-expansion cohort (120 mg/m²) in the phase I/II RAINFOL-01 study (NCT05579366) (56). These results have provided strong impetus for the initiation of phase III studies in OC and EC (RAINFOL-02/03). Rina-S has not received FDA approval and is currently in phase I/II evaluation.

### 6.2 HER2

HER2 overexpression has been associated with adverse clinicopathologic features and poorer outcomes in subsets of OC, although its prognostic significance remains less consistent than in breast cancer (74). HER2 overexpression is an established indicator of adverse prognosis in OC and is closely associated with disease progression and unfavorable outcomes. In the 40-patient OC cohort of DESTINY-PanTumor02, T-DXd achieved an ORR of 45%, with rates of 63.6% in the HER2 IHC 3+ subgroup and 36.8% in the IHC 2+ subgroup; the median PFS was 5.9 months and the median OS was 13.2 months (3). NCCN guidelines specify that T-DXd is an option for patients with recurrent OC (HER2 IHC 3+/2+) (38). T-DXd (Enhertu^®^) received FDA accelerated approval in April 2024 for a pan-tumor indication encompassing HER2-positive (IHC 3+) unresectable or metastatic OC (as well as CC and EC). The ongoing phase III DESTINY-Ovarian01 trial is evaluating T-DXd plus bevacizumab versus bevacizumab alone as first-line maintenance therapy in patients with HER2-expressing OC, exploring its role in earlier treatment settings.

### 6.3 Trop2

Multiple Trop2-targeted ADCs are currently being positioned across the gynecologic oncology landscape, with Sac-TMT being the first to enter phase III evaluation (the TroFuse series). In the multicenter phase II KL264–01 study (NCT04152499), single-agent Sac-TMT achieved an ORR of 40.0% (37.1% in the platinum-resistant subgroup), a median PFS of 6.0 months, and a DCR of 75.0% in 40 patients with OC who had progressed after prior platinum-based therapy (63). TROPION-PanTumor03 (NCT05489211) reported an ORR of 42.9% (34.6% in the platinum-resistant subgroup), a median PFS of 5.6 months, and a DCR of 75.0% with Dato-DXd in 35 patients with high-grade serous or endometrioid OC who had received 1–2 prior platinum-based regimens (60). SHR-A1921 and DB-1305 have demonstrated comparable efficacy (ORR 41.4%–58.8%). All of the above Trop2-targeted ADCs (Sac-TMT, Dato-DXd, etc.) have not received FDA approval for OC indications and are currently in phase II/III clinical evaluation.

### 6.4 Other antigens

Beyond FRα, HER2, and Trop2, a succession of ADCs targeting other antigens are actively undergoing clinical research in OC. The publicly accessible research targets encompass CDH6 (cadherin 6), B7-H4, B7-H3, CLDN6 (claudin 6), mesothelin, and NaPi-IIb, among others.

Raludotatug deruxtecan (R-DXd, DS-6000) is a CDH6-targeted ADC with a DAR of 8 and is loaded with a topoisomerase I inhibitor (DXd). In a Phase I dose-escalation and expansion study (NCT04707248) (75), previously treated OC patients exhibited promising anti-tumor activity. In the 4.8-8.0 mg/kg dose cohort, the median DOR was 5.8 months, the DCR was 88.9%, and the median PFS was 8.1 months.

Multiple B7-H4 targeted ADCs are being assessed in early clinical studies. Puxitatug Samrotecan (AZD8205) is a B7-H4-targeted ADC (DAR 8) developed by AstraZeneca. In a phase I/II study (NCT05123482) (75), the ORR of OC patients was 3/17 (17.6%). Emiltratug ledadotin (XMT-1660) is another B7-H4 - targeted ADC (DAR 6). In its first-in-human study (NCT05377996), the overall ORR of B7-H4 expressing solid tumor patients was 31%. Additionally, GSK5733584 (NCT06069557) and HS-20089 (NCT05263479) have SGN-B7H4V a preliminarily reported ORR of 4/20 (20%) in the OC cohort. Currently, a phase III clinical study of GSK7733584 (NCT07286331) has been initiated, yet recruitment has not commenced. (NCT05194072) has an ORR of 2/3 in a small-sample OC cohort; however, due to the extremely small sample size, more experimental data are required for future support.

Apart from CDH6 and B7-H4, ADCs targeting CLDN6, B7-H3, mesothelin, and NaPi-IIb are also in preclinical or early clinical development at various stages. Among them, the NaPi-IIb - targeted ADC upifitamab rilsodotin (XMT-1536) has been evaluated in a phase I extended study (NCT03319628) for platinum-resistant OC patients, demonstrating preliminary anti-tumor activity. The CLDN6 targeted ADC has drawn attention due to the high-frequency expression of claudin 6 in ovarian serous cancer; however, clinical data remain limited at present.

It should be noted that the ADCs targeting CDH6, B7-H4, and other emerging targets mentioned above are all in the early (phase I/II) stages of clinical research, and none of them have received approval from the FDA. A comprehensive assessment of their efficacy confirmation, optimal patient selection strategy, and safety characteristics still requires validation through larger randomized controlled trials to provide sufficient evidence for their inclusion in the standard treatment regimen for OC.

## 7 Management of ADC-related toxicities

In the era of precision medicine, in addition to its own advantages, ADC also needs to pay special attention to its drug toxicity, mainly focusing on eye toxicity, pulmonary toxicity, liver toxicity, hematological and neurological toxicity. Some ADCs, such as those containing microtubule inhibitor MMAF, may cause corneal lesions, manifested as blurred vision, photophobia, corneal epithelial damage, etc. Taking TV as an example, the incidence of grade ≥ 3 eye adverse reactions in the innovatTV301 study was approximately 11% (25). Therefore, standardized prevention and monitoring of eye toxicity should be emphasized before and during treatment. It is recommended to undergo ophthalmic examinations, including visual assessment and slit lamp examination, and corneal fluorescein staining if necessary, before the first administration and before each treatment cycle. In terms of prevention, it is recommended that patients regularly use preservative free artificial tears and, if necessary, combine them with corticosteroid eye drops under the guidance of an ophthalmologist; for TV treatment, measures such as cold compress and vasoconstrictive eye drops can also be taken during infusion to reduce eye exposure. Patients should avoid wearing contact lenses and be advised to seek medical attention promptly if symptoms such as blurred vision, eye pain, photophobia, or foreign body sensation occur. For patients who have experienced corneal toxicity, medication should be temporarily suspended, treatment delayed, or dosage adjusted according to the severity. Through preventive eye care, early identification, and timely intervention, most ADC-related eye toxicity can be effectively controlled, thereby reducing the risk of permanent visual impairment (25).

Interstitial lung disease (ILD) is a serious adverse event requiring heightened vigilance in gynecologic patients receiving DXd-containing ADC therapy. In the DESTINY-PanTumor02 study of T-DXd, the incidence of ILD/pneumonitis was approximately 12.1%, with grade ≥3 events occurring in 3.0% of patients (3). Because a subset of patients with gynecologic cancers may have baseline respiratory compromise due to advanced disease burden, pulmonary metastases, pleural effusion, prior radiotherapy, or previous systemic therapies, early recognition of ILD is particularly important. Given that gynecologic cancer patients frequently present with baseline respiratory compromise secondary to the underlying malignancy or prior therapies, early recognition of ILD is particularly important. The emergence of dry cough, dyspnea, or hypoxemia during treatment should prompt immediate high-resolution computed tomography (HRCT) and joint consultation between oncology and pulmonology services. Corticosteroids constitute the first-line treatment for ILD; severe cases may require hospitalization for intravenous methylprednisolone pulse therapy, with periodic follow-up of pulmonary function and thoracic imaging (76).

Hepatotoxicity principally manifests as transaminase elevation. MMAE-containing ADCs are almost universally associated with some degree of hepatotoxicity; in select studies, the incidence of grade ≥3 hepatic enzyme elevations has been approximately 5%–10% (77). Accordingly, routine liver function testing prior to treatment initiation and before each cycle is warranted, with hepatoprotective agents administered when clinically indicated (78).

Hematologic and neurologic toxicities exert a substantial impact on the quality of life of gynecologic cancer patients. Peripheral neuropathy is a well-recognized adverse effect of ADCs carrying tubulin inhibitor payloads and may result in extremity numbness, pain, and functional impairment, adversely affecting daily activities and treatment adherence (79). ASCO guidelines recommend duloxetine for the management of chemotherapy-induced neuropathic pain and advocate for multidisciplinary collaboration to adjust treatment regimens (80). Hematologic toxicities — including neutropenia (grade ≥3 incidence approximately 16%–30%), thrombocytopenia, and anemia — necessitate regular complete blood count monitoring and timely administration of growth factor support or transfusion therapy (81). Comprehensive management of these toxicities is essential not only for maintaining treatment continuity but also for optimizing patient quality of life.

## 8 Drug resistance mechanism of ADC

ADC resistance typically represents a multifaceted and hierarchical process that can manifest during the stages of target binding, intracellular transport, drug release, and drug action (82). Tumor cells can mitigate the effective binding and endocytosis of ADCs through downregulating the expression of target antigens, antigen loss, or augmenting intratumoral expression heterogeneity, thereby attenuating the therapeutic response (83, 84). For example, downregulation or loss of HER2 expression has been identified as a key resistance mechanism to trastuzumab emtansine (T-DM1), and similar antigen-dependent escape has been observed with other ADC targets (83, 85). Following cellular entry, ADCs must undergo processes such as receptor-mediated endocytosis, endosome-to-lysosome trafficking, and intracellular proteolytic processing. Consequently, reduced endocytosis efficiency, impaired endosomal–lysosomal trafficking, or defective intracellular processing may result in inadequate release of the active payload (86, 87). Alterations in caveolin-1-mediated endocytic pathways, deficiency of lysosomal transporter SLC46A3, and changes in lysosomal pH have all been implicated in resistance to ADCs bearing maytansinoid payloads (86, 88). Payload-related drug resistance is also a crucial mechanism. Particularly, when drug efflux pumps such as ATP-binding cassette subfamily B member 1 (ABCB1)/multidrug resistance protein 1 (MDR1) and multidrug resistance-associated protein 1 (MRP1) are upregulated, the intracellular accumulation of microtubule inhibitors or other efflux-susceptible cytotoxins can be substantially diminished (89, 90). Moreover, adaptive alterations in the downstream action pathway of the payload by tumor cells, such as modifications in β-tubulin isoform expression affecting microtubule dynamics, upregulation of anti-apoptotic proteins (e.g., BCL-2 family members), or enhanced DNA damage repair capacity, may further compromise ADC cytotoxic activity (91, 92). From the perspective of drug design, insufficient linker stability or poor intracellular drug release efficiency can lead to “functional resistance” (82, 93). The former may cause premature payload release in the circulation, increase off-target toxicity, and reduce the effective payload delivered to the tumor, while the latter restricts the liberation of active cytotoxins within tumor cells (93, 94). In summary, ADC resistance is not attributable to a single mechanism but rather results from the combined effects of antigen-dependent, intracellular transport-dependent, and payload-dependent mechanisms (82, 83). In the future, drug resistance may be delayed or overcome by optimizing target selection (including bispecific targeting to mitigate antigen loss), enhancing linker chemistry and conjugation techniques, employing payloads less susceptible to efflux pumps (such as topoisomerase I inhibitors), and developing dual-payload or bispecific ADCs (95).

## 9 Future perspectives

As ADC conjugation technologies continue to evolve, next-generation ADC exploration is primarily focused on refining one or more of the constituent components. Several novel ADC formats have emerged: bispecific ADCs (BsADCs) can engage different epitopes on the same antigen or two distinct antigens to address the challenges of tumor heterogeneity and resistance; dual-payload ADCs (DpADCs) carry two cytotoxic payloads with complementary mechanisms of action, producing synergistic antitumor effects; peptide-drug conjugates (PDCs) employ peptides to achieve efficient tumor site targeting and payload release, substantially reducing off-target effects; and radionuclide-drug conjugates (RDCs) integrate radionuclides into targeting ligands, enhancing both precision delivery and potent cytotoxic activity.

In the domain of combination strategies, the co-administration of ADCs with immune checkpoint inhibitors, poly (ADP-ribose) polymerase (PARP) inhibitors, and antiangiogenic agents is under active investigation and holds the potential to further augment therapeutic efficacy. For example, MIRV is currently being explored in combination with bevacizumab (NCT02606305). At the same time, the combination of MIRV and immunotherapy is also underway, such as NCT03835819 evaluating its efficacy and tolerability in combination with pembrolizumab in FRα-positive recurrent OC. The combination of Trop2 targeted ADC Dato-DXd and immune checkpoint inhibitors has also entered early research in pan tumor types. For example, TROPION-PanTumor03 (NCT05489211) is evaluating the application of Dato-DXd combined with durvalumab, carboplatin, and other regimens in advanced solid tumors, including the gynecological tumor extension cohort.

Precision biomarker selection represents another critical future direction, encompassing the standardization of target expression assays (e.g., harmonized scoring systems for HER2, FRα, and Trop2), genomic characterization (e.g., homologous recombination deficiency (HRD) status, microsatellite instability (MSI)/mismatchrepair (MMR) status), and the elucidation of resistance-associated molecular mechanisms — all of which will facilitate the individualized and precision-guided application of ADC therapy. Liquid biopsy, as a non-invasive and resampling detection method, has shown potential application value in drug resistance monitoring and efficacy evaluation of ADC therapy. By detecting circulating tumor DNA (ctDNA), molecular changes associated with ADC resistance can be dynamically tracked, such as downregulation or mutation of target antigen encoding genes, amplification or upregulation of drug efflux pump related genes (such as ABCB1), and resistance mutations related to the loading pathway (96, 97). In addition, phenotype analysis of circulating tumor cells (CTCs) can provide real-time information on target antigen protein levels, which can help evaluate the dynamic evolution of antigen heterogeneity during treatment and provide a basis for the continued use or regimen adjustment of ADCs (98). In the field of gynecological tumors, liquid biopsy has been preliminarily applied for molecular typing and treatment monitoring of OC, EC, etc., but its systematic research in the context of ADC therapy is still limited (99). In the future, integrating liquid biopsy into the full management of ADC therapy is expected to achieve early identification of drug resistance mechanisms, dynamic assessment of target antigen status, and personalized treatment decisions based on molecular features. However, its clinical practicality still needs further prospective research to verify.

Next-generation ADCs will be engineered to address the shortcomings of existing agents, optimizing each constituent component to enhance stability and targeting while reducing cytotoxicity, thereby realizing truly efficacious, safe, and personalized cancer treatment and improving outcomes in gynecologic malignancies.
