# Supplementary material for: Antibody-drug conjugates in gynecologic malignancies: breakthroughs, challenges, and future directions
Source: Front Immunol. 2026 Jun 15;17:1831465. doi: 10.3389/fimmu.2026.1831465 (PMC13310996; doi:10.3389/fimmu.2026.1831465)
Supplement: Supplementary file 1 [file Table1.docx]

**Supplementary Table 1. Antibody–Drug Conjugates Investigated in Gynecologic Malignancies by Molecular Target**

| **Target** | **ADC (Generic Name)** | **Antibody** | **Linker Type** | **Payload** | **Regulatory Status** |
| --- | --- | --- | --- | --- | --- |
| **FRα** | Mirvetuximab soravtansine | Humanized anti-FRα IgG1 | Cleavable (sulfo-SPDB) | DM4 (maytansinoid) | **FDA approved(2022)** |
|  | Luveltamab tazevibulin | Anti-FRα | Cleavable | SC209 (hemiasterlin) | Investigational |
|  | Rinatabart sesutecan | Farletuzumab | Cleavable | Eribulin | Investigational |
|  | GSK5733584 | Anti-FRα | Undisclosed | Undisclosed | Investigational |
| **HER2** | Trastuzumab emtansine | Trastuzumab | Non-cleavable (SMCC) | DM1 (maytansinoid) | Approved (breast); investigational (gyn) |
|  | Trastuzumab deruxtecan | Trastuzumab | Cleavable (GGFG) | DXd (Topo I inhibitor) | FDA approved  (breast) |
|  | Trastuzumab duocarmazine | Trastuzumab | Cleavable (vc) | Duocarmycin (seco-DUBA) | Investigational |
|  | Disitamab vedotin | Disitamab | Cleavable (mc-vc-PABC) | MMAE | investigational |
|  | BNT323/DB-1303 | Anti-HER2 (novel) | Cleavable (tetrapeptide) | DXd (Topo I inhibitor) | Investigational |
|  | ARX788 | Anti-HER2 | Non-cleavable | Amberstatin269 | Investigational |
|  | MRG002 | Anti-HER2 | Cleavable (mc-vc-PABC) | MMAE | Investigational |
|  | A166 | Anti-HER2 | Cleavable | Duo-5 (maytansinoid derivative) | Investigational |
|  | Zanidatamab zovodotin | Zanidatamab (biparatopic) | Cleavable | Auristatin (ZD02044) | Investigational |
|  | TAA013 | Anti-HER2 | Non-cleavable (SMCC) | DM1 (maytansinoid) | Investigational |
| **TROP2** | Sacituzumab govitecan | Sacituzumab | Cleavable (CL2A) | SN-38 (Topo I inhibitor) | Approved (breast); investigational (gyn) |
|  | Datopotamab deruxtecan | Anti-TROP2 | Cleavable (GGFG) | DXd (Topo I inhibitor) | Investigational |
|  | Sacituzumab tirumotecan | Anti-TROP2 | Cleavable | Belotecan derivative (Topo I) | Investigational |
|  | Lorlatuzumab vedotin | Anti-TROP2 | Cleavable (mc-vc-PABC) | MMAE | Investigational |
| **Tissue Factor (TF)** | Tisotumab vedotin | Tisotumab | Cleavable (mc-vc-PABC) | MMAE | **FDA approved** (2021) |
| **B7-H4** | Puxitatug samrotecan | Anti-B7-H4 | Cleavable | Topo I inhibitor | Investigational |
|  | Emiltatug ledadotin | Anti-B7-H4 | Cleavable (Dolaflexin) | AF-HPA (auristatin) | Investigational |
|  | SGN-B7H4V | Anti-B7-H4 | Cleavable (mc-vc-PABC) | MMAE | Investigational |
|  | HS-20089 | Anti-B7-H4 | Undisclosed | Undisclosed | Investigational |
| **CDH6** | Raludotatug deruxtecan | Anti-CDH6 | Cleavable (GGFG) | DXd (Topo I inhibitor) | Investigational |
| **NaPi2b** | Upifitamab rilsodotin | Anti-NaPi2b | Cleavable (Dolaflexin) | AF-HPA (auristatin) | Investigational |
|  | Lifastuzumab vedotin | Anti-NaPi2b | Cleavable (mc-vc-PABC) | MMAE | Discontinued |
| **Mesothelin** | Anetumab ravtansine | Anti-mesothelin | Cleavable (SPDB) | DM4 (maytansinoid) | Investigational |
|  | BMS-986148 | Anti-mesothelin | Cleavable | Tubulysin | Investigational |
| **CLDN6** | XB002/AZD0901 | Anti-CLDN6 | Cleavable | Undisclosed | Investigational |
| **B7-H3** | Ifinatamab deruxtecan | Anti-B7-H3 | Cleavable (GGFG) | DXd (Topo I inhibitor) | Investigational |
|  | Vobramitamab duocarmazine | Anti-B7-H3 | Cleavable (vc) | Duocarmycin (seco-DUBA) | Investigational |
| **MUC16 (CA-125)** | Sofituzumab vedotin | Anti-MUC16 | Cleavable (mc-vc-PABC) | MMAE | Discontinued |
|  | DMUC4064A | Anti-MUC16 | Non-cleavable (SMCC) | DM4 (maytansinoid) | Discontinued |
| **Nectin-4** | Enfortumab vedotin | Anti-Nectin-4 | Cleavable (mc-vc-PABC) | MMAE | Approved (urothelial); investigational (gyn) |

**Abbreviations:** OC, ovarian cancer; CC, cervical cancer; EC, endometrial cancer; DAR, drug-to-antibody ratio; DM1/DM4, maytansinoid derivatives; DXd, deruxtecan; MMAE, monomethyl auristatin E; SN-38, topoisomerase I inhibitor; Topo I, topoisomerase I; vc, valine-citrulline; PABC, para-aminobenzylcarbamate; SMCC, succinimidyl-4-(N-maleimidomethyl)cyclohexane-1-carboxylate; SPDB, N-succinimidyl-4-(2-pyridyldithio)butanoate; GGFG, glycine-glycine-phenylalanine-glycine.
